# Acute Neuromuscular Adaptations in the Postural Control of Patients with Parkinson’s Disease after Perturbed Walking

**DOI:** 10.3389/fnagi.2017.00316

**Published:** 2017-09-27

**Authors:** Cristian F. Pasluosta, Simon Steib, Sarah Klamroth, Heiko Gaßner, Julia Goßler, Julius Hannink, Vinzenz von Tscharner, Klaus Pfeifer, Juergen Winkler, Jochen Klucken, Bjoern M. Eskofier

**Affiliations:** ^1^Digital Sports Group, Pattern Recognition Lab, Department of Computer Science, Friedrich-Alexander University Erlangen-Nürnberg, Erlangen, Germany; ^2^Laboratory for Biomedical Microtechnology, Department of Microsystems Engineering, University of Freiburg, Freiburg, Germany; ^3^Institute of Sport Science and Sport, Friedrich-Alexander University Erlangen-Nürnberg, Erlangen, Germany; ^4^Department of Molecular Neurology, University Hospital Erlangen, Friedrich-Alexander University Erlangen-Nürnberg, Erlangen, Germany; ^5^Human Performance Laboratory, University of Calgary, Calgary, AB, Canada

**Keywords:** neuromuscular, postural control, treadmill intervention, center-of-pressure, dynamics, Parkinson

## Abstract

Patients suffering from Parkinson’s disease (PD) present motor impairments reflected in the dynamics of the center of pressure (CoP) adjustments during quiet standing. One method to study the dynamics of CoP adjustments is the entropic half-life (EnHL), which measures the short-term correlations of a time series at different time scales. Changes in the EnHL of CoP time series suggest neuromuscular adaptations in the control of posture. In this study, we sought to investigate the immediate changes in the EnHL of CoP adjustments of patients with PD during one session of perturbed (experimental group) and unperturbed treadmill walking (control group). A total of 39 patients with PD participated in this study. The experimental group (*n* = 19) walked on a treadmill providing small tilting of the treadmill platform. The control group (*n* = 20) walked without perturbations. Each participant performed 5-min practice followed by three 5-min training blocks of walking with or without perturbation (with 3-min resting in between). Quiet standing CoP data was collected for 30 s at pre-training, after each training block, immediately post-training, and after 10 min retention. The EnHL was computed on the original and surrogates (phase-randomized) CoP signals in the medio-lateral (ML) and anterior–posterior (AP) directions. Data was analyzed using four-way mixed ANOVA. Increased EnHL values were observed for both groups (Time effect, *p* < 0.001) as the intervention progressed, suggesting neuromuscular adaptations in the control of posture. The EnHL of surrogate signals were significantly lower than for original signals (*p* < 0.001), confirming that these adaptations come from non-random control processes. There was no Group effect (*p* = 0.622), however by analyzing the significant Group by Direction by Time interaction (*p* < 0.05), a more pronounced effect in the ML direction of the perturbed group was observed. Altogether, our findings show that treadmill walking decreases the complexity of CoP adjustments, suggesting neuromuscular adaptations in balance control during a short training period. Further investigations are required to assess these adaptations during longer training intervals.

## Introduction

Parkinson’s disease (PD) is the most common progressive neurodegenerative disorder after Alzheimer’s disease, with a reported incidence between 410 and 529 per 100,000 person-years in the elderly (Wirdefeldt et al., 2011). Patients with PD present motor impairments with the cardinal symptoms bradykinesia, tremor, rigidity, and postural instability (Jankovic, 2008). These motor impairments are the results of disruptions in the neuromuscular control of movements and they are reflected in the dynamics of neuromuscular motor outputs such as center of pressure (CoP) adjustments during quiet standing (Schmit et al., 2006).

The dynamics of CoP adjustments over time have been analyzed using several non-linear methods in healthy young and elderly populations (Manor et al., 2010; Baltich et al., 2014, 2015), and in patients with different neurological diseases (Stergiou and Decker, 2011; Negahban et al., 2013). One aspect of the dynamics of CoP adjustments is its complexity, which has been shown to decrease with aging and disease (Schmit et al., 2006), to decrease after repeated induced effort by walking (Louis et al., 2015), and to increase after long-term physical interventions (Manor et al., 2013; Chen and Jiang, 2014; Wayne et al., 2014). Thus, studying the dynamics of CoP adjustments during quiet standing provides insights on the patient neuromuscular state and may assist in disease diagnosis and in the assessment of therapy outcomes.

One particular method to study the dynamics of CoP adjustments over time is the entropic half-life (EnHL). The EnHL measures the short-terms correlations existing in CoP time series at different time scales (Zandiyeh and Von Tscharner, 2013; Baltich et al., 2014). More specifically, it is defined as the time scale at which current CoP adjustments are no longer correlated with previous ones. Thus, increased EnHL values (i.e., when CoP adjustments are correlated to previous ones in shorter time scales) correspond to less regular signals, with lower levels of determinism, and lower complexity. The EnHL is related to others well-known multi-scale measures such as de-trended fluctuation analysis (von Tscharner et al., 2016), with the advantage of a meaningful physical interpretation as it provides a measure in units of time. The EnHL of CoP adjustments can be influenced by several characteristics of the neuromuscular postural control system. For example, a decrease in the EnHL was observed if more mechanical degrees of freedoms are present to stabilize a specific movement, but it may also increase if the frequency of the control system interventions adjusting the CoP motion increase (Federolf et al., 2015). Further, the interplay of multiple control strategies suggest multiple control pathways, usually referred as the neuromuscular solution space. A larger solution space could be one reason for an increasing complexity of CoP adjustments over time and would decrease the EnHL. Finally, by randomizing the structure encoded in the phase of the CoP time series (i.e., phase-randomized surrogates) and comparing the EnHL of this phase-randomized surrogates with the EnHL of the original CoP time series, it is possible to conclude whether the fluctuations in the CoP adjustments are a result (or not) of a non-random neuromuscular control process (Enders et al., 2014).

In a previous work, we observed that patients suffering from PD presented immediate adaptations in their gait patterns after receiving a single session of perturbed treadmill walking (Klamroth et al., 2016). No postural control adaptations were observed for patients undergoing perturbed walking, although patients walking without perturbations presented a moderated increase in CoP area during quiet standing (Klamroth et al., 2016). However, it is still not clear whether no postural control adaptations were present in the perturbed group or whether the measures used in this study (i.e., traditional average measures of CoP area and velocity) were unable to capture these adaptations.

Therefore, in this study we sought to investigate the dynamics of the CoP adjustments assessed by the EnHL to further infer the immediate neuromuscular adaptations in the postural control of patients with PD during and after these interventions. Changes in the EnHL during or after the intervention would suggest immediate adaptations in the neuromuscular postural control of patients. These adaptations may reflect either a change in the neuromuscular solution space or changes in the intervention-rate of the postural control system. Importantly, a higher regularity (i.e., longer EnHL values) in the CoP time series compared to their phase-randomized surrogates would confirm the non-random nature of these neuromuscular adaptations.

## Materials and Methods

### Participants

The participants and group allocation design was described in previous work (Klamroth et al., 2016). Briefly, a total of 39 patients with PD were recruited in the Movement disorder unit, Department of Molecular Neurology of the University Hospital Erlangen, Erlangen, Germany. Participants were randomly assigned to either a perturbation group (*n* = 19) or a control group (*n* = 20). The participants’ characteristics are summarized in **Table 1**. Inclusion criteria encompassed being diagnosed with PD (following the guidelines of the German Society for Neurology), Hoehn and Yahr (H&Y) disease stage (Goetz et al., 2004) between 1 and 3.5, Unified Parkinson’s Disease Rating Scale (UPDRS-III) subscore ‘gait’ and/or ‘postural instability’ equal or greater than 1 (Goetz et al., 2007), and the ability to walk without assistance. Participants diagnosed with other neurological diseases, and with cardiovascular or orthopedic disorders were excluded from this study. Participant signed an informed consent, which was approved by the local ethics committee of the Medical Faculty, Friedrich-Alexander University Erlangen-Nürnberg (FAU). Erlangen, Germany (IRB-approval-Re. -No. 4208. 21.04.2010). All subjects gave written informed consent in accordance with the Declaration of Helsinki. The protocol was approved by the Medical Faculty, Friedrich-Alexander University Erlangen-Nürnberg (FAU). Erlangen, Germany.

**Table 1:** Patient characteristics (mean ± standard deviation; ^†^data from *n* = 27; ^††^data from *n* = 38; ^∗^*p*-value < 0.05), modified with permission from Klamroth et al. (2016).

	Perturbation (*n* = 19)	Control (*n* = 20)	*p*-Value
Age (years)	64.8 ± 10.3	64.2 ± 8.5	0.833
Gender (male/female)	11/8	18/2	0.022^∗^
Weight (kg)	74.9 ± 13.0	83.2 ± 13.7	0.060
Height (cm)	173.9 ± 8.0	175.2 ± 13.7	0.731
H&Y (0-5)	2.4 ± 0.6	2.2 ± 0.9	0.476
UPDRS-III	16.7 ± 5.5	17.7 ± 8.7	0.662
Disease duration (years)^†^	7.3 ± 4.2	6.9 ± 4.6	0.791
Number of falls in the past 12 months (%)^††^			0.106
0	7	9	
1	3	8	
2	5	0	
= > 3	4	2	
Levodopa-equivalent daily dose	591.47 ± 262.21	524.28 ± 269.46	0.436

*UPDRS-III values refer to meds-on condition. None freezing episodes nor dyskinesia were observed during the time of this study.*

### Treadmill Intervention

**FIGURE 1:**
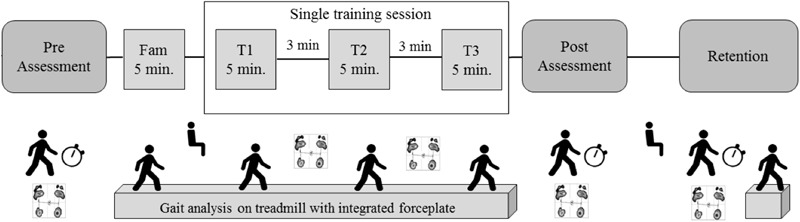
Study protocol, adapted from Klamroth et al. (2016), with permission.

The intervention protocol was described in detail in a previous work (Klamroth et al., 2016). Briefly, the perturbation group walked on a standard medical treadmill (mercury, h/p/cosmos medical GmbH) mounted on a tiltable platform (zebris Medical GmbH), which provided small three-dimensional tilting movements to the treadmill platform. The control group walked on the same treadmill without perturbations. The training protocol was identical for both groups (**Figure 1**). First, participant adapted to walk on the treadmill during a 5-min familiarization block (the perturbation group experimented perturbation walking in the last minute). Immediately after treadmill familiarization, participants performed three 5-min blocks of treadmill walking at 70% of their self-selected overground walking speed with or without perturbation, with 3 min of resting (standing) in between blocks.

CoP data was collected for 30 s (sampled at 100 Hz) during quiet stance with eyes open using an integrated pressure sensor matrix (FDM-T, zebris Medical GmbH, Isny, Germany) at pre-training, after each 5-min walking blocks (T1, T2), immediately post-training, and after 10 min of retention. A total of three trials were performed at pre-training, one trial at T1 and T2, two trials post-training, and two trials after retention.

### Data Analysis

Center of pressure data was analyzed for 29 s after the first 0.3 s of each CoP measurement. First, the CoP data in both medio-lateral (ML) and anterior–posterior (AP) directions were band-pass filtered using a wavelet filter (von Tscharner and Schwameder, 2001) with cut-off frequencies of 0.15 and 10 Hz. In addition, phase-randomized surrogates of the CoP data were computed using the amplitude adjusted Fourier transform (AAFT) method (Theiler et al., 1992). The EnHL was then calculated on the filtered CoP and surrogates data following the methodology described in previous work (Baltich et al., 2015; Federolf et al., 2015). In short, it consisted of gradually randomizing the signals at different time scales using the reshape scale method (Zandiyeh and Von Tscharner, 2013). For each randomization step, the fuzzy sample entropy (*m* = 3, *r* = 0.7, exponent = 0.5) was then computed and normalized with respect to its maximum value, which was obtained by a total randomization of the time series. The EnHL was then determined as the time scale required to reach half the maximum entropy, representing the time scale at which the signal switches from a deterministic behavior to a random one. The exact EnHL was computed by linear interpolation. A total of 45 rescales (corresponding to timescale between 10 and 450 ms) were computed for each CoP and surrogate signal. The EnHL values were averaged across trials. Data analysis was performed in Matlab version R2014a (The MathWorks, Inc., Natick, MA, United States).

### Statistical Analysis

A four-way mixed ANOVA was implemented for the EnHL values calculated from the original and surrogate CoP data to test for between-subject factors of *Group* (two levels, perturbation and control), *Direction* (two levels, ML and AP), *Signal* (two levels, original and surrogates), and to test for within-subject factor of *Time* (five levels, pre-training, T1, T2, post-training, and retention). *Post hoc* tests with Bonferroni corrections were conducted in the case of significant interaction. Statistical significance was set at the α level of 0.05. The statistical analysis was performed using R (R Core Team, 2016).

## Results

**FIGURE 2:**
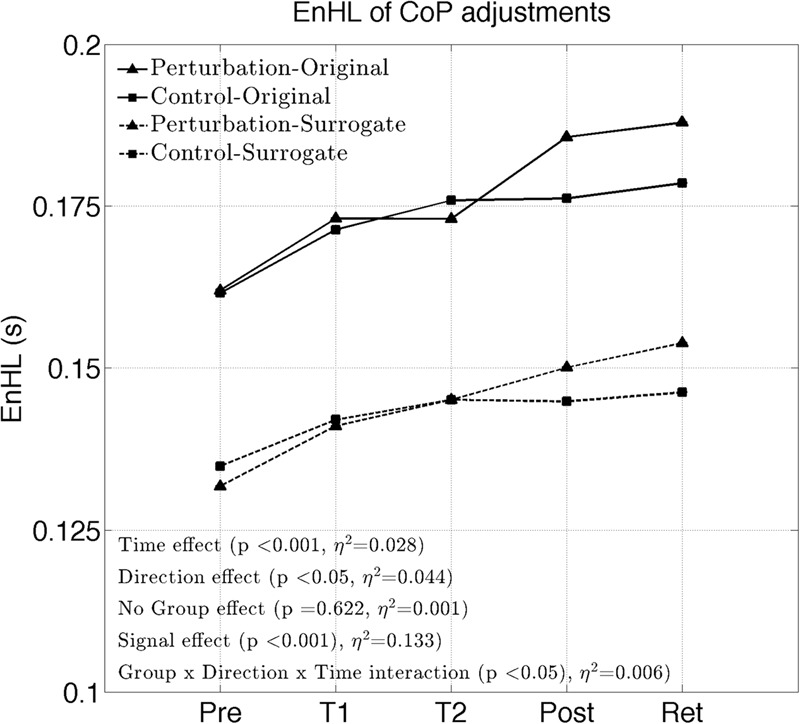
Changes of the EnHL during and after treadmill intervention.

The EnHL of the original CoP signals significantly increased with time (significant *Time* effect, **Figure 2** and **Table 2**). No significant *Group* effect or *Group* × *Time* interaction were present (*F* = 0.24, *p* = 0.622). A significant *Direction* effect was also observed (*F* = 9.82, *p* < 0.05). A significant *Group* × *Direction* × *Time* interaction was present (*F* = 2.90, *p* < 0.05). The EnHL of the surrogates were significantly lower than the EnHL of the original CoP signals (significant *Signal* effect, **Figure 2** and **Table 2**).

**Table 2:** Results of four-way mixed ANOVA.

	*F*	*p*-Value	η^2^
Group	0.245	0.622	0.001
**Signal**	**32.695**	**<0.001**	**0.133**
**Direction**	**9.828**	**0.002**	**0.044**
**Time**	**13.828**	**<0.001**	**0.028**
Group × Signal	0.029	0.864	0.000
Group × Direction	0.630	0.429	0.003
Signal × Direction	1.609	0.207	0.008
Group × Time	1.467	0.211	0.003
Signal × Time	0.313	0.869	0.001
Direction × Time	1.765	0.134	0.004
Group × Signal × Direction	0.061	0.806	0.000
Group × Signal × Time	0.120	0.975	0.000
**Group** × **Direction** × **Time**	**2.903**	**0.021**	**0.006**
Signal × Direction × Time	0.446	0.776	0.001
Group × Signal Direction × Time	0.201	0.938	0.000

*Bold font represents significance at the α ≤ 0.05 level.*

**FIGURE 3:**
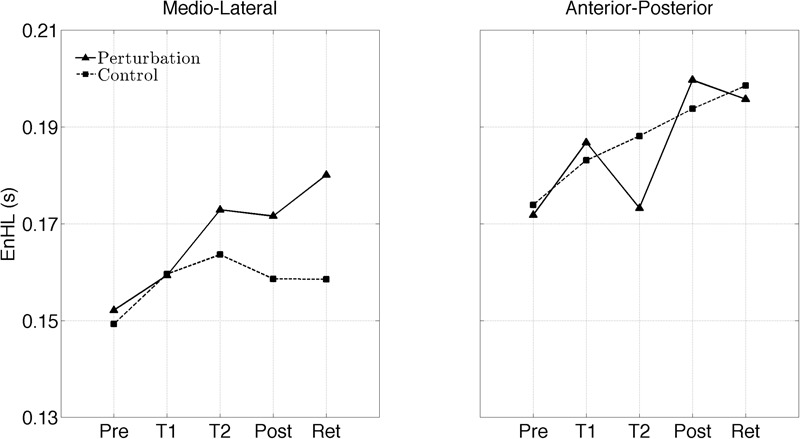
Changes of the EnHL during and after treadmill intervention for the ML **(left)** and AP **(right)** directions of the original CoP data.

The interaction plots of **Figure 3** (original CoP signals) show different slopes for the perturbation and control group in the ML direction, while similar slopes are observed for both groups in the AP direction. This interaction was further analyzed using *post hoc* tests. *Post hoc* tests revealed a significant difference (*p* < 0.05) in EnHL values between pre-training and retention for the perturbation group in the ML direction (**Tables 3, 4**). None of the other comparisons were significant at the α level of 0.05.

**Table 3:** Results of the *post hoc* tests for the EnHL values of the original CoP signals of the perturbation group (μ: mean; SE: standard error).

Perturbation – original					
**Direction**	**Time**		**μ_I_–μ_J_ (ms)**	***SE* (ms)**	***p*-Value**
	**I**	**J**			

AP	Pre	T1	-15.00	-3.40	1
		T2	-1.44	0.39	1
		Post	-27.91	-3.31	0.191
		Retention	-23.96	-0.85	0.130
	T1	T2	13.56	3.80	1
		Post	-12.90	0.09	1
		Retention	-8.96	2.55	1
	T2	Post	-26.46	-3.71	0.122
		Retention	-22.52	-1.25	0.375
	Post	Retention	3.94	2.46	1
ML	Pre	T1	-7.22	0.48	1
		T2	-20.76	-5.30	0.613
		Post	-19.47	-0.22	0.191
		Retention	**-27.95**	**-2.54**	**0.025**
	T1	T2	-13.54	-5.79	1
		Post	-12.25	-0.71	1
		Retention	-20.73	-3.02	0.497
	T2	Post	1.29	5.08	1
		Retention	-7.19	2.76	1
	Post	Retention	-8.49	-2.32	1

*Bold font represents significance at the α ≤ 0.05 level.*

**Table 4:** Results of the *post hoc* tests for the EnHL values of the original CoP signals of the control group (μ: mean; SE: standard error).

Control – original					
**Direction**	**Time**		**μ_I_–μ_J_ (ms)**	***SE* (ms)**	***p*-Value**
	**I**	**J**			

AP	Pre	T1	-9.22	-1.51	1
		T2	-14.27	-1.90	0.379
		Post	-19.89	-0.93	0.268
		Retention	-24.67	0.77	0.087
	T1	T2	-5.05	-0.40	1
		Post	-10.68	0.57	0.926
		Retention	-15.45	2.28	0.845
	T2	Post	-5.63	0.97	1
		Retention	-10.40	2.68	1
	Post	Retention	-4.78	1.70	1
ML	Pre	T1	-10.36	0.36	1
		T2	-14.36	0.49	0.178
		Post	-9.35	0.36	0.607
		Retention	-9.27	1.59	0.687
	T1	T2	-4.00	0.13	1
		Post	1.01	0.01	1
		Retention	1.09	1.23	1
	T2	Post	5.01	-0.12	1
		Retention	5.09	1.10	1
	Post	Retention	0.08	1.22	1

## Discussion

The purpose of this work was to study immediate neuromuscular adaptations in postural control of patients suffering from PD during a single session of perturbed treadmill walking. We found a reduced complexity in the adjustments of their CoP during quiet standing as the intervention progressed. While no significant *Group* effect was present, the perturbed group demonstrated more pronounced intervention effects in the ML direction (*Group* × *Direction* × *Time* interaction), coinciding with the direction in where the treadmill was mostly perturbed. The results also showed a more regular structure of the CoP adjustments in both groups compared to their phase-randomized surrogates, confirming that the observed neuromuscular adaptations emerged from non-random control processes.

The EnHL adds to the battery of available non-linear methods developed to capture the dynamics of CoP adjustments produced by the postural control system. The changes in the dynamics of the CoP adjustments observed in this study provide further insights on the immediate neuromuscular adaptations occurring during short periods of training time, which could not be observed using traditional sway measures such as CoP area and velocity. Thus, this study provides further evidence on the valuable information contained in the complex variability of CoP adjustments, complementing traditional sway measures.

Increased EnHL values over the course of the treadmill intervention may reflect a continued reduction of the neuromuscular solution space, suggesting a decrease in the number of control pathways stabilizing the standing posture. Longer EnHL values also imply that the CoP adjustments are related to previous ones for longer periods of time and thus suggest less frequent interventions of the postural control on the CoP motion. A higher level of determinism was observed in the CoP adjustments of female participants after two 6-min treadmill walking blocks without perturbations (Louis et al., 2015). The authors interpreted this reduced complexity of the CoP adjustments as a possible change in the hip strategy affected by the effort induced during walking. Again and in line with this interpretation, the longer EnHL values observed over and after the treadmill intervention may reflect a reduced neuromuscular solution space, allowing less randomness in the neuromuscular motor control of posture as an immediate effect induced by the effort of walking on the treadmill.

A higher complexity in the CoP adjustments over time has been shown to be correlated with healthy populations (Manor and Lipsitz, 2013). Further, an increase in the complexity of CoP adjustments was observed after 24 weeks of Tai Chi training in healthy older adults (Wayne et al., 2014) and in older adults with peripheral neuropathy (Manor et al., 2013), as well as after 12 weeks of resistance training in patients with cardiovascular disease, diabetes mellitus and osteoporosis (Chen and Jiang, 2014). In our study, we observed the opposite effect after one session of perturbed walking. Because of the findings reported in the literature, the results of our study may be transitory and these immediate neuromuscular adaptations may reverse, producing more complex CoP adjustments after several weeks of training as observed in healthy populations.

Even though, the effects between the intervention and control group on the EnHL values were not significantly different (i.e., no main effect of *Group*), a significant *Group* × *Direction* × *Time* interaction was observed (**Table 2** and **Figure 3**). Further *post hoc* analyses revealed a significant difference between the EnHL measured at retention from the values measured at pre-training in the ML direction of the perturbation group. This was the only comparison reaching statistical significance, suggesting that only the perturbed treadmill induced immediate neuromuscular adaptations in the postural control of patients, particularly in the ML direction. Further, the observation of shorter EnHL values in the ML direction compared to the AP direction (**Figure 3**) may be attributed to the mechanical degrees of freedom involved in postural control as the ML direction provides a larger range of possible mechanical configurations compared to the AP direction. This effect was also observed during quiet bipedal stance in a healthy young population (Federolf et al., 2015).

A limitation of this study is the lack of an experimental condition with closed eyes due to protocol constraints. Postural adjustments during the eyes closed condition may differ from eyes open condition as patients with PD may compensate impaired sensorimotor functions by relying more on visual feedback. Further investigations should focus on evaluating immediate changes of perturbed walking in the complexity of CoP adjustments during the eyes closed condition. The lack of a healthy control group prevents the direct comparison of the EnHL values measured in patients with PD against healthy individuals. Involving a control group may help in understanding how the adaptations observed during each treatment compare to healthy populations. Finally, the proportion of female and male participants in this study was statistically different between the two groups (*p* = 0.022, **Table 1**). This bias in gender composition could contribute to the observed differences in dynamics of CoP adjustments, which warrants further investigation in a larger and gender-balanced cohort of participants.

## Conclusion

We observed non-random immediate neuromuscular adaptations after one session of perturbed treadmill walking, which were reflected in changes in EnHL of CoP adjustments. These findings add to our previous work (Klamroth et al., 2016), where no postural control adaptations were observed with traditional measures of CoP area and velocity. Even though, other non-linear methods were not evaluated in this study, the results suggest that measuring the non-random complex nature of the CoP adjustments prove useful to study adaptations in postural control during quiet standing. The complexity of a time series can be measured with other non-linear methods such as de-trended fluctuation analysis. The EnHL values provide additional information as they are measured in units of time, which may be useful to understand the time scales governing postural control strategies. Further studies should be conducted to analyze the long-term effect of perturbed treadmill walking on the neuromuscular postural control in patients suffering from PD.

## Author Contributions

CP performed data analysis, designed and performed statistical analysis, and analyzed the results. SS, SK and HG, designed the study protocol, performed data collection, and helped analyzing the results. JG helped performing data collection. JH, VVT, KP, JW, JK, and BE helped analyzing the results. VVT developed the EnHL measure. All the authors helped preparing the manuscript.

## Conflict of Interest Statement

The authors declare that the research was conducted in the absence of any commercial or financial relationships that could be construed as a potential conflict of interest.
